# Giant group I intron in a mitochondrial genome is removed by RNA back-splicing

**DOI:** 10.1186/s12867-019-0134-y

**Published:** 2019-06-01

**Authors:** Sylvia Ighem Chi, Mikael Dahl, Åse Emblem, Steinar D. Johansen

**Affiliations:** 1 Department of Medical Biology, Faculty of Health Sciences, UiT–The Arctic University of Norway, Tromsø, Norway; 2 Genomics Group, Faculty of Biosciences and Aquaculture, Nord University, Bodø, Norway

**Keywords:** *Amplexidiscus*, Back-splicing, Catalytic RNA, Group I intron, Intron retention, Mitochondrial RNA, Ribozyme, *Ricordea*

## Abstract

**Background:**

The mitochondrial genomes of mushroom corals (Corallimorpharia) are remarkable for harboring two complex group I introns; ND5-717 and COI-884. How these autocatalytic RNA elements interfere with mitochondrial RNA processing is currently not known. Here, we report experimental support for unconventional processing events of ND5-717 containing RNA.

**Results:**

We obtained the complete mitochondrial genome sequences and corresponding mitochondrial transcriptomes of the two distantly related corallimorpharian species *Ricordea yuma* and *Amplexidiscus fenestrafer*. All mitochondrial genes were found to be expressed at the RNA-level. Both introns were perfectly removed by autocatalytic splicing, but COI-884 excision appeared more efficient than ND5-717. ND5-717 was organized into giant group I intron elements of 18.1 kb and 19.3 kb in *A. fenestrafer* and *R. yuma*, respectively. The intron harbored almost the entire mitochondrial genome embedded within the P8 peripheral segment.

**Conclusion:**

ND5-717 was removed by group I intron splicing from a small primary transcript that contained a permutated intron–exon arrangement. The splicing pathway involved a circular exon-containing RNA intermediate, which is a hallmark of RNA back-splicing. ND5-717 represents the first reported natural group I intron that becomes excised by back-splicing from a permuted precursor RNA. Back-splicing may explain why Corallimorpharia mitochondrial genomes tolerate giant group I introns.

**Electronic supplementary material:**

The online version of this article (10.1186/s12867-019-0134-y) contains supplementary material, which is available to authorized users.

## Background

Hexacorallia represents an important subclass of marine cnidarians that hosts several well-known orders like Scleractinia (stony corals), Actiniaria (sea anemones), Zoantharia (colonial anemones), and Corallimorpharia (mushroom corals). Corallimorpharia appears closely related to Scleractinia, but if they represent true naked corals is currently under debate [1, 2]. The order Corallimorpharia consists of about 50 valid species organized into seven families [3]. They are solitary polyps that usually occur in larger groups in tropical habitats, and morphologically they have short tentacles and no stony skeletons [3].

The mitochondrial genomes (mtDNAs) of species representing most hexacorallian orders have been sequence characterized, and subsequently applied in phylogenetic analyses and molecular studies [4–7]. The mtDNAs are vertebrate-like, which include a small-sized (17–22 kb) circular organization with a high gene density [8]. They harbor the same set of ribosomal RNA (rRNA) genes and the 13 protein-coding genes of the oxidative phosphorylation (OxPhos) system as most metazoan mitochondrial genomes, but optional genes may occur in some species [7–11]. Hexacoral mtDNA contains a highly reduced tRNA gene repertoire, and all the conventional mitochondrial genes are encoded by the same DNA strand [8, 12]. The most remarkable feature, however, is the presence of autocatalytic group I introns [4, 13].

Group I introns are intervening sequences that encode self-splicing ribozymes responsible for the RNA processing reactions [14]. Intron sequences are removed from the precursor RNA in a two-step transesterification pathway that involves RNA cleavage and ligation activities [15]. Splicing is initiated by a nucleophilic attach at the 5′ splice site (SS) by a noncoded guanosine cofactor (exo-G). This reaction leads to covalent attachment of exo-G to the released intron’s 5′ end. In a subsequent reaction, the free 3′ end of the upstream exon attacks the 3′ SS, resulting in exon ligation and release of the linear intron RNA. The catalytic property of group I intron RNA is funded on a highly organized and complex three-dimensional ribozyme structure that consists of a catalytic domain, a folding domain, and a substrate domain [16, 17]. Each domain contains a conserved subset of secondary structure paired elements (P1 to P9), where P7 harbors the catalytic core structure designed to bind different guanosine factors in Step 1 (exo-G) and Step 2 (ɷG) of the splicing reaction [14].

All hexacoral species and isolates investigated to date harbor a group I intron in the mitochondrial NADH dehydrogenase 5 (ND5) gene at position 717 [4]. This obligatory intron (ND5-717) is strictly vertically inherited, and appears to have a fungal origin [4]. One unusual feature of hexacoral ND5-717 is the large insertion in one of its ribozyme paired segments (P8) that includes several cognate mitochondrial gene sequences, from two genes in Actiniaria and Zoantharia [7, 10] to 16 genes in Corallimorpharia [1, 5]. The mitochondrial cytochrome oxidase I (COI) gene contains different, but optional, group I introns at positions 720, 867, and 884 [10, 18, 19]. Whereas COI-720 and COI-867 appeared confined to the Indo-Pacific scleractinians [20] and to zoantharians [10], respectively, COI-884 is more widely distributed in species belonging to several hexacorallian orders. All COI introns contain homing endonuclease gene (HEG) sequences, encoding a LAG-type (LAGLIDADG) homing endonuclease [18, 21].

Corallimorpharian mitochondrial genomes are slightly larger in size (20 to 22 kb) compared to other hexacorallian orders and contain two group I introns, ND5-717 and COI-884 [1, 5]. We have previously noted a highly complex organization of ND5-717 in Corallimorpharia that involves 16 genes embedded in P8, including the COI-884 group I intron containing COI gene [17]. Recently, we reported RNAseq-based mitochondrial transcriptome (mito-transcriptome) profiling in several actiniarian and zoantharian species [7, 10, 11]. Different from the Corallimorpharia, these anemones harbor only two mitochondrial genes (ND1 and ND3) embedded within P8 of their respective ND5-717 introns. We found all mitochondrial protein genes to be expressed at the RNA level, and gained supporting evidence that ND5-717, COI-867, and COI-884 introns were perfectly spliced out from their RNA precursors [7, 10, 11].

Here, we have characterized the mito-transcriptomes of the two distantly related corallimorpharian species *Ricordea yuma* and *Amplexidiscus fenestrafer*. All mitochondrial genes were found expressed, and their introns (ND5-717 and COI-884) were perfectly removed by RNA splicing. We further investigated the ND5 mRNA precursor and the ND5-717 intron RNA splicing intermediate, and concluded that the intron was excised by back-splicing. Back-splicing is relatively common in vertebrate spliceosomal introns, resulting in exon permutation and covalently closed circular RNAs [22–24]. However, the ND5-717 introns in *R. yuma* and *A. fenestrafer* are the first reported examples of group I intron back-splicing.

## Results

### Mitochondrial genome sequences of *R. yuma* and *A. fenestrafer*

**Fig. 1:**
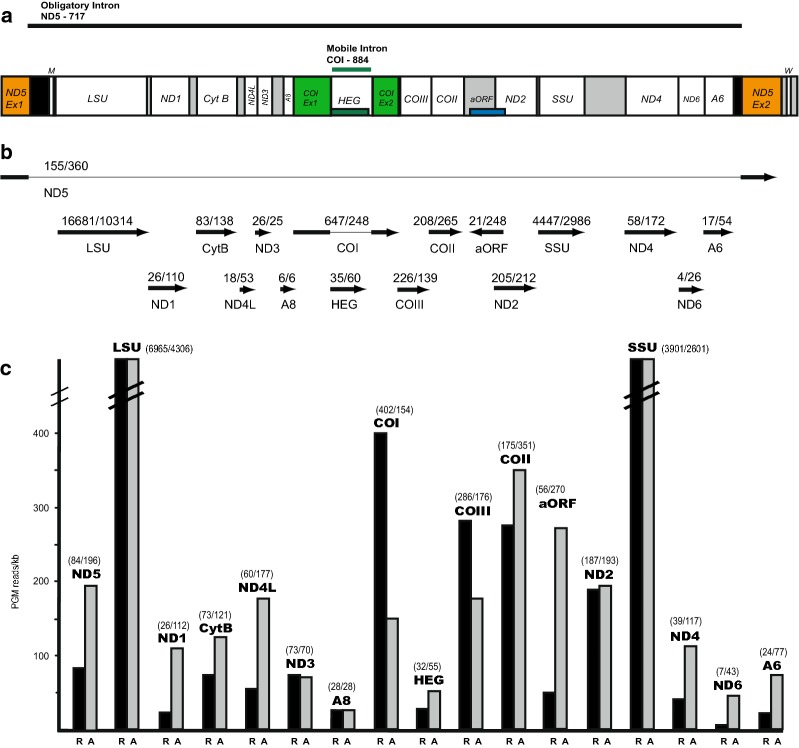
Mitochondrial genome and transcripts in *Ricordea yuma* and *Amplexidiscus fenestrafer*. **a** Schematic view of mitochondrial genome content and organization of circular mtDNA (presented as a linear map). Obligatory ND5-717 and mobile-like COI-884 introns are indicated. *ND5* exons and *COI* exons are shown in orange and green colors, respectively. Abbreviations: *SSU* and *LSU*, mitochondrial small- and large-subunit ribosomal RNA genes. *ND1*-*6*, NADH dehydrogenase subunit 1 to 6 genes. *COI*-*III*, cytochrome c oxidase subunit I to III genes. *A6* and *A8*, ATPase subunit 6 and 8 genes. *CytB*, cytochrome B gene; *HEG*, homing endonuclease gene; *aORF*, antisense open reading frame. *W* and *M*; tRNA genes for Trp and Met, respectively, indicated by the standard one-letter symbols for amino acids. **b** Mapped transcript reads generated by Ion Torrent PGM from protein coding and rRNA coding regions. Presented below is read coverage per gene region (*R. yuma*/*A. fenestrafer*). **c** Histograms representing estimated normalized read numbers for *R. yuma* (R; black) and *A. fenestrafer* (A; grey). Three separate PGM runs were performed for each species. The PGM transcript number of each RNA was normalized to the size of gene coding regions (PGM reads/kb). The estimated normalized read numbers vary between approximately 7 reads/kb and 6965 reads/kb

The circular mitochondrial genomes of *R. yuma* (21,430 bp) and *A. fenestrafer* (20,054) were determined on both strands using a combined strategy of Ion Torrent PGM (average coverage of 126× and 70×, respectively) and Sanger sequencing. All canonical mitochondrial genes were coded on the same strand and in a similar order compared to those of most corallimorpharian mtDNAs investigated (Fig. 1a) [1, 5]. In addition to the conserved set of 13 OxPhos protein coding genes, *R. yuma* and *A. fenestrafer* encoded a homing endonuclease and an antisense open reading frame (aORF) transcript. Several notable sequence features related to the protein-coded genes were observed. Firstly, a significant fraction (approximately 40%) of the reading frames contain a GTG initiation codon (Additional file 1: Table S1), suggesting that tRNA-fMet recognizes both ATG and GTG at this position. This feature was conserved among all corallimorpharian mtDNAs [5]. Secondly, group I introns were present at conserved positions (884 and 717) within the COI and ND5 genes, respectively. Thirdly, the ND5-717 was noted as a giant intron in *R. yuma* (19.3 kb) and *A. fenestrafer* (18.9 kb), harboring almost the complete mtDNA sequence between its 5′ and 3′ splice sites (Fig. 1a). Finally, COI-884 was identified as a 1.2 kb group I intron containing a LAG-type HEG.

We recognized 18 intergenic regions (IGRs) in the *R. yuma* and *A. fenestrafer* mtDNAs (Additional file 1: Table S1). Most IGRs were found to be identical, or almost identical, in size among the corallimorpharian mtDNAs available in the NCBI database. We found the *R. yuma* mtDNA to be 1376 bp larger than that of *A. fenestrafer*, and this size difference was mainly due to IGR-5, IGR-7, IGR-11 and IGR-13. No recognizable molecular features or signatures could be assigned to size heterogeneities in IGR-5 and IGR-7. The most dramatic variation was present within IGR-13. While *R. yuma* contained a 1132 bp IGR-13, *A. fenestrafer* had a sequence corresponding to only 431 bp. The extra sequences were mainly due to two direct repeat motifs (123 bp and 87 bp), both represented by two imperfect copies.

### ORF transcript at the opposite strand

IGR-11 was found in two size variants, represented by 928 bp in *R. yuma* and 541 bp in *A. fenestrafer* (Additional file 1: Table S1). We noted that the 5′ region of ND2 gene was extended corresponding to a putative N-terminal domain highly conserved among corallimorpharians. Similar extensions were recently reported in several zoantharian mitochondrial genes [10], suggesting that the current mitochondrial gene annotation in hexacorals needs revision. The main feature, however, of IGR-11 was an ORF present on the opposite strand in *R. yuma* and *A. fenestrafer* (Fig. 1a). Homologous antisense ORF (aORF) sequences were present in all corallimorpharian mtDNAs investigated, but at different size variants (Additional file 2: Figure S1). Interestingly, a large part of the aORF was antisense to the ND2 gene, and this antisense region was significantly longer in *A. fenestrafer* than *R. yuma*. While the *R. yuma* aORF corresponded to a protein of 125 amino acids, the aORF protein in *A. fenestrafer* was significantly larger (306 amino acids). BLAST analysis did not reveal any significant similarity to known proteins in databases. The *A. fenestrafer* and *R. yuma* aORF regions were found to be transcribed, but at different levels (see below).

### Mitochondrial transcriptome analysis

RNAseq mapping analysis of Ion Torrent PGM sequenced reads was used to assess the mitochondrial transcripts. About 7.6 million and 4.7 million quality-filtered reads of total poly(A) RNA in *R. yuma* and *A. fenestrafer*, respectively, were generated. We unambiguously identified 23,394 reads (0.31%) and 16,690 reads (0.35%) as mitochondrial transcripts in *R. yuma* and *A. fenestrafer* (Fig. 1b). When normalizing the reads numbers against gene sizes (Fig. 1c), several interesting and relevant observations were noted. (1) rRNA transcripts were about 5 to 20 times more abundant compared to most of the mRNA transcripts. (2) All protein coding mRNAs in both species were represented by multiple reads. A general trend was that Complex IV transcripts were more abundant than transcripts of Complex I and Complex V, which corroborates mito-transcriptome analyses in actiniarians and zoantharians [7, 10, 11]. (3) COI-884 encoded homing endonuclease transcripts were found despite the fact that no recognizable initiation codons could be recognized, and that no in-frame fusions with the upstream COI-exon could be detected. (4) The proposed 5′ extension of ND2 gene was transcribed at same level as the main part of the gene. This indicated that ND2 gene is significantly longer than previously annotated [1, 5] corroborating recent observations in zoantharians [10]. (5) The longest version of aORF present in *A. fenestrafer*, but not that in *R. yuma*, was expressed at the same RNA level as Complex IV transcripts. This may indicate a biological role in the mitochondria. (6) Transcripts corresponding to perfectly ligated exons of ND5 and COI mRNAs were identified, and supported group I intron splicing activities of ND5-717 and COI-884.

### RNA splicing and processing of ND5-717 and COI-884 group I introns

**Fig. 2:**
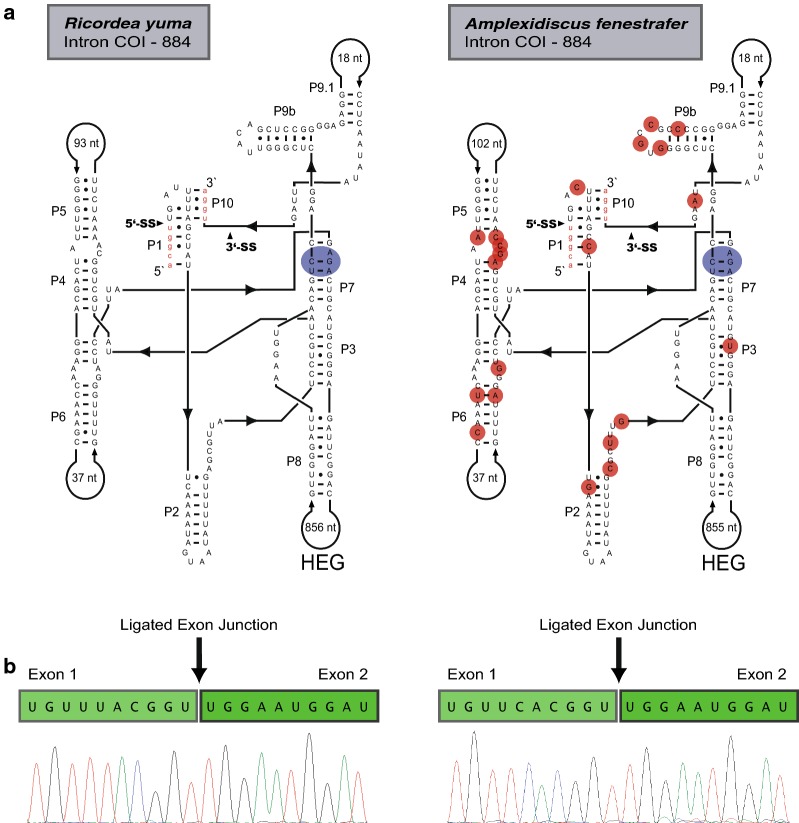
COI gene features in *Ricordea yuma* and *Amplexidiscus fenestrafer*. **a** Secondary structure diagrams of corallimorpharian COI-884 group I introns (*R. yuma*, left; *A. fenestrafer*, right). The ten conserved paired segments of the catalytic core (P1 to P10) are shown, and flanking COI exons sequences are in red letters. The P8 extension containing the HEG is indicated and in small red circles are nucleotides substitutions in *A. fenestrafer* compared to *R. yuma*. The blue circles show the catalytic core sequences at the G-binding site. **b** Representation of COI mRNA sequences consisting of ligated exons. The ligation junctions are indicated, and below are the sequencing chromatograms

**Fig. 3:**
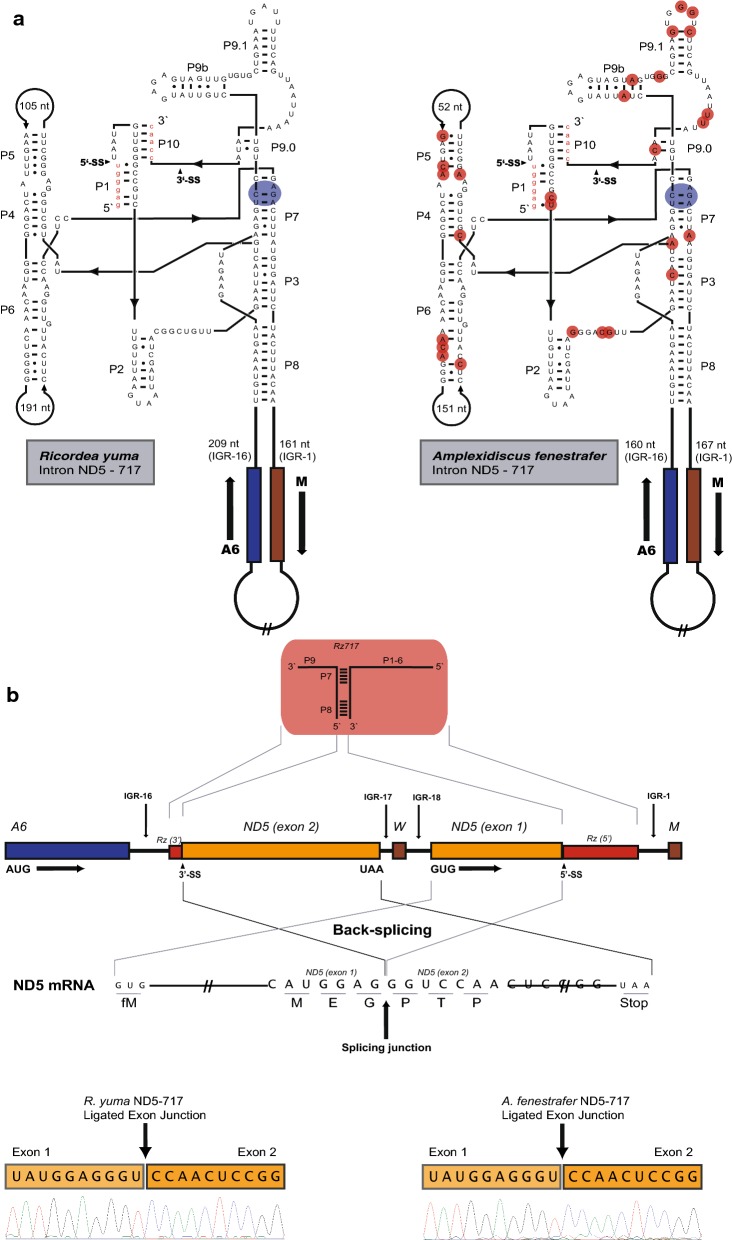
Mitochondrial ND5 gene organization and ND5-717 intron secondary structure in *Ricordea yuma* and *Amplexidiscus fenestrafer*. **a** Secondary structure diagrams of ND5-717 group I introns in *R. yuma* (left) and *A. fenestrafer* (right). The ten conserved paired segments of the catalytic core (P1 to P10) are shown and flanking ND5 exons sequences are in red letters. The P8 extensions containing mitochondrial genes are indicated. The blue circles indicate catalytic core sequences at the G-binding site and in small red circles are nucleotides substitutions in *A. fenestrafer* compared to *R. yuma*. **b** Organization map of mitochondrial ND5 gene in Corallimorpharian. A schematic view of the ND5-717 ribozyme is presented above the map. The splice sites are distant apart, but brought in proximity in a permuted intron–exon order due to the circular organization of the mtDNAs. Thus, ND5 exon 2 is presented upstream of ND5 exon 1. ND5 mRNA consisting of the ligated exons with the splicing junction indicated is shown below. Chromatograms of the exon ligation sequences are shown for *R. yuma* (left) and *A. fenestrafer* (right)

RNA secondary structure diagrams were made according to the general model of the group I intron RNA core [16, 25] as well as available comparative data on hexacoral mitochondrial group I introns [4, 7]. Diagrams of the ND5-717 and COI-884 group I intron ribozymes revealed similar catalytic cores and peripheral regions (Figs. 2a, 3a) compared to that of other hexacorallian orders [4, 7, 10, 11]. All intron RNAs contained large insertions in P8, and in COI-884 this insertion consisted of a HEG. This homing endonuclease ORF was extended beyond P8 and into the ribozyme core, both at the 5′ and 3′ ends, and thus organized similarly as those in actiniarians [7, 11]. The P8 insertion in ND5-717 was extensive and contained two ribosomal rRNA genes, one tRNA gene, 12 OxPhos genes, the COI-884 and its HEG, and the transcribed antisense ORF (Fig. 1a). The P8 insertion is the main reason for the giant size of ND5-717.

We found ND5-717 and COI-884 to generate perfectly ligated RNA exons in vivo (Figs. 2b, 3b), despite the fact that ND5-717 lacked the universal conserved terminal G-residue (ɷG) essential for second step in splicing (Fig. 3a). Relative splicing efficiency of COI-884 and ND5-717 was estimated from transcriptome data by assessing the observed fractions of un-spliced and spliced exon sequences. We found a similar tendency in *R. yuma* (Additional file 3: Figure S2A–D) and *A. fenestrafer* (Additional file 4: Figure S3A–D). Ligated exons appeared > 10 times more abundant for COI mRNA than ND5 mRNA. While the majority (> 70%) of ND5 precursor mRNAs were non-spliced and linked to flanking intron sequences, nearly 100% of the COI mRNA exons were perfectly ligated (Additional file 5: Table S2). The observation was supported by RT-qPCR (Additional file 5: Table S2). We conclude that splicing of COI-884 appeared more efficient compared to ND5-717.

Next, we investigated the ability of the introns to generate RNA circles in vivo. We used an RT-PCR/Sanger sequencing approach with inverted intron-specific primers designed to generate amplicons from circular intron RNA [26–28]. Circularization of ND5-717 was predicted to generate giant RNA circles corresponding to 19.3 kb and 18.0 kb in *R. yuma* and *A. fenestrafer*, respectively. However, no circular intron junctions were detected for ND5-717 (Additional file 3: Figure S2F, Additional file 4: Figure S3F); a result not surprising due to the large circular RNA sizes and the fact that ND5-717 lacked the essential ɷG for the circularization mechanism [29]. Interestingly, intron RNA circles were detected in COI-884 (Additional file 3: Figure S2E, Additional file 4: Figure S3E). All circles appeared to involve the 3′ intron sequence (including ɷG), but Sanger sequencing indicated more than one donor site at the intron 5′ end in addition to full-length intron circles both in *R. yuma* (Additional file 3: Figure S2G) and *A. fenestrafer* (Additional file 4: Figure S3G).

### ND5-717 intron RNA is removed by back-splicing

Group I introns are usually removed from precursor RNAs by conventional *cis*-splicing. However, conventional splicing of ND5-717 is extremely challenging due to the extensive P8 insertion, and the requirement of a single unprocessed precursor RNA of approximately 20 kb. Based on the assumption that individual RNA processing of all the 16 embedded mitochondrial genes have to be totally repressed prior to ND5-717 splicing, and the fact that COI-884 intron splicing was found significantly more efficient than ND5-717, we reasoned that conventional splicing was highly unlikely. RNAseq mapping of the complete mito-transcriptome in both *R. yuma* and *A. fenestrafer* indicated distinct transcription units, which further supports this notion (data not shown).

**Fig. 4:**
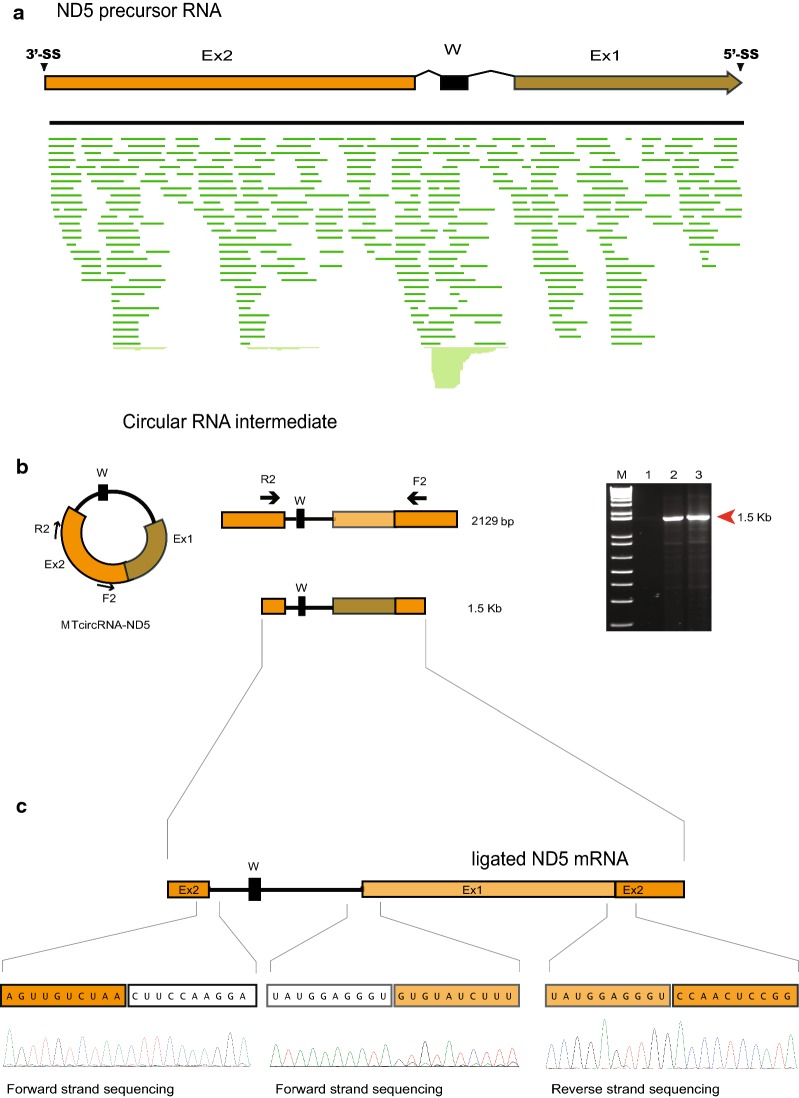
RNA back-splicing by ND5-717 group I intron in corallimorpharians. **a** In silico mapping of Ion PGM transcriptome reads to the ND5 precursor RNA. The *R. yuma* map is shown. The map also corresponds to the 2.13 kb circular RNA intermediate (MTcircRNA-ND5). Ex1, ND5 exon 1; Ex2, ND5 exon 2; W, tRNA Trp. **b** Map of circular RNA intermediate. Primers F2/R2 binds to exon 2, but in opposite directions. Right panel—the 1.5 kb F2/R2 amplicon from *R. yuma* (lane 2) and *A. fenestrafer* (lane 3). M, 1 kb ladder marker; lane 1, negative control. **c** Chromatogram of the 1.5 kb amplicon (exemplified in *R. yuma*)

Consequently, two alternative splicing modes were considered to explain ND5-717 splicing; *trans*-splicing and back-splicing. Whereas *trans*-splicing depends on two separate primary transcripts, a single primary transcript with permuted intron–exon arrangement is involved in back-splicing. It is important to note that, due to the circular organization of the mitochondrial genome, ND5 exon 2 is located upstream and adjacent to exon 1 (Fig. 3b). RNAseq mapping supported the existence of a continuous permuted exon transcript in *R. yuma* (Fig. 4a) and *A. fenestrafer* (Additional file 6: Figure S4). Furthermore, *trans*-splicing and back-splicing can be unambiguously distinguished through a circular RNA intermediate (2.1 kb), which is only generated through back-splicing (Fig. 4b).

To search for a back-splicing circular hallmark, we used the inverted exon primer approach, followed by RT-PCR, plasmid cloning, and Sanger sequencing. In this approach primers were designed to specifically bind to ND5 exon 2 sequences (primer set F2/R2), but in opposite orientations compared to regular PCR amplification (Fig. 4b, left panel). Amplicons were only generated if this exon (exon 2) was ligated to exon 1, and located on a circular RNA species. Indeed, amplicons of expected sizes (1.5 kb) were generated in *R. yuma* and *A. fenestrafer* (Fig. 4b, lanes 2 and 3 in right panel). Sequencing of plasmid-cloned amplicons unambiguously showed that the ND5 exon 2 stop codon (UAA), the ND5 exon 1 initiation codon (GUG), and the ND5 exon ligation site (AGGGU/CCAACU) were all present in the same continuous sequence (Fig. 4c). These results strongly supported a circular RNA splicing intermediate, a finding consistent with ND5-717 back-splicing.

## Discussion

We have provided experimental support for unconventional RNA back-splicing of a group I intron in mtDNAs of two distantly related corallimorpharian species. Furthermore, the mtDNAs contain a previously undetected ORF located at the opposite strand compared to all other mitochondrial genes. This aORF contains highly conserved amino acid sequences among corallimorpharian species, and is expressed at the RNA level. If the aORF RNA becomes translated in the mitochondria, or exerts its function at the RNA level (long non-coding RNA/antisense RNA) is currently not known.

### Intron back-splicing in mitochondrial gene expression

The obligatory ND5-717 in hexacoral mtDNA is unique. It carries multiple mitochondrial genes inserted into P8, has a highly compact conserved catalytic core secondary structure, and terminates with ɷA instead of the universally conserved ɷG as in most group I introns [4]. These unusual characteristics of ND5-717 justify a more detailed investigation related to mode of splicing, splicing efficiency, and influence on mitochondrial transcription and RNA processing.

ND5-717 has a highly compact and conserved catalytic core secondary structure with similarities to most model group I ribozymes [4, 17]. This suggests that the intron folds into catalytically competent conformation for active site formation. RNAseq mapping data supports biological activity of ND5-717, and the RT-PCR/sequencing approach generates ligated ND5 exons showing that the intron was correctly spliced. However, due to the large size of ND5-717 that includes almost the entire mitochondrial genome in corallimorpharians, we questioned its mode of splicing. For this intron to perform conventional *cis*-splicing, processing of all the gene sequences within P8 have to be completely repressed until the intron is removed. This possibility was considered highly unlikely since the mito-transcriptome expressed a discontinuous transcript map indicating strong and efficient processing sites, or distinct transcription units. Consequently, we evaluated *trans*-splicing or back-splicing as more plausible alternatives to explain the ligated exon fragments.

Only a few mitochondrial group I intron have been reported to *trans*-splice in bacteria, protozoans, fungi or plants [30–33], including designed constructs for therapeutic applications [34, 35]. Natural occurring group I intron back-splicing has not been reported, but the mitochondrial ND5-717 intron in the deep-water sea anemone *Protanthea simplex* is suggested to be removed by back-splicing [36]. However, artificial back-splicing of engineered group I introns are known from the well-studied *Anabaena* pre-tRNA and *Tetrahymena* introns. These examples were based on permuted intron–exon self-splicing approach that produced circular RNAs [37–39]. For spliceosomal introns, however, back-splicing appears to be common, at least in vertebrates, giving rise to new classes of large non-coding RNAs [22–24]. A hallmark of back-splicing is the presence of an essential circular RNA intermediate. We detected a corresponding circular intermediate (MTcircRNA-ND5) in *R*. *yuma* and *A*. *fenestrafer*. We provide support that the giant ND5-717 intron in corallimorpharians splices via back-splicing, but from a much shorter permuted precursor RNA. Nonetheless, *trans*-splicing was not excluded as a co-mechanism for the removal of ND5-717.

### Is intron retention a regulatory feature of ND5-717 splicing?

That exon and intron sequences at splice sites can influence self-splicing activity is well established for group I intron [14, 29, 30]. Moreover, a single nucleotide difference at conserved sites may result in intron repression, and subsequently suppress exon splicing [40, 41]. We reported the splicing efficiency of ND5-717 to be reduced compared to that of COI-884 in the same mitochondrial genome. This observation suggested ND5-717 retention. In mammals, the ND5 gene appears as the only mitochondrial OxPhos gene that is tightly regulated at the RNA level and probably influences respiration [42, 43]. Among other potential functions, exonic circRNAs may act as regulators of mRNA levels or translation [28, 44, 45]. Similarly, formation of ND5 exonic circular RNA (MTcircRNA-ND5) by ND5-717 back-splicing could potentially regulate the level of mature ND5 mRNA in hexacorals, and thus the overall respiration rate. Likewise, retention of ND5-717 could play a regulatory role in ND5 mRNA maturation and mitochondrial transcription [7].

## Conclusion

Giant group I introns of almost 20,000 nt in size require an extraordinary RNA splicing approach. We provide experimental support that the ND5 mRNA exons in corallimorpharian mitochondria are ligated by RNA back-splicing, catalyzed by the ND5-717 group I intron ribozyme. A surprisingly short primary transcript (approximately 3000 nt) contains a permutated intron–exon arrangement where ND5 exon 2 is followed by ND5 exon 1. A consequence of group I intron splicing is that the ND5 exons are joined in the correct order. Interestingly, the large P8 insertion in ND5-717 does not apparently interfere with mRNA splicing, explaining why the giant group I intron might be tolerated in mitochondria. ND5-717 represents the first reported group I intron that catalyzes back-splicing from a natural permuted precursor RNA.

## Methods

### Nucleic acid isolation

*Ricordea yuma* and *A. fenestrafer* specimens were purchased from Akvariemagasinet Oslo, Norway in 2012 (http://www.akvariemagasinet.no/) and kept alive in our lab reef tank at the Arctic University of Norway (UiT) until nucleic acid isolations. Approximately 20 mg of fresh tissue polyps was used separately for DNA and RNA isolations based on Wizard Genomic DNA Purification (Promega, Madison, WI, USA) and Trizol Reagent (ThermoFisher Scientific, Waltham, MA, USA) manuals, respectively. Genomic DNA was obtained by homogenizing the tissue in 600 µl nuclei lysis solution (Promega, Madison, WI, USA) for 10 s at 5000 rpm using Precellys 24 homogenizer (Stretton Scientific, Stretton, UK). Phenol–chloroform extraction was performed to eliminate polysaccharide contaminants, followed by elution of the purified DNA in Nuclease-Free water (ThermoFisher Scientific, Waltham, MA, USA). A pre-extraction step involving crushing of tissue in liquid nitrogen was performed prior to total RNA isolation. The tissue was then homogenized in Trizol Reagent using the above Precellys settings. Purified RNA was obtained by repeated phenol–chloroform extraction, precipitation in isopropanol at 4 °C overnight, followed by two washing steps in 70% ethanol, and finally resolved in nuclease-free water.

### Ion Torrent PGM sequencing

Next generation sequencing of both DNA and RNA was achieved using Ion Personal Genomic Machine (Ion Torrent PGM) platform in our lab at UiT. Ion Torrent manufacturer’s procedures were followed for library construction, template preparation and deep sequencing. Approximately 1 µg genomic DNA, sheared at optimized setting of 30 s on Covaris S2 system (Covaris, Woburn, MA, USA), was used for 300 bp fragment library construction. DNA library preparation was then completed using Ion Xpress™ Plus gDNA Fragment Library Preparation Kit and protocol (ThermoFisher Scientific, Waltham, MA, USA). Template dilution factor was determined based on qPCR analysis from KAPA Library Quantification procedure (KAPA BIOSYSTEMS, Wilmington, MA, USA). A 200 bp cDNA library was constructed from 8 µg total RNA based on Ion Total RNA-Seq Kit procedure. The RNA was poly(A)-enriched using mRNA DIRECT Purification Kit (ThermoFisher Scientific, Waltham, MA, USA), fragmented enzymatically, hybridized, Ion adapter ligated, cDNA synthesized, and finally amplified. The DNA and cDNA libraries were independently enriched on Ion OneTouch System and sequenced using Ion 316 chips in four separate runs on Ion PGM.

### Mitochondrial genome assembly and sequence annotation

The complete mitogenomes of *R. yuma* and *A. fenestrafer* were assembled from 3.1 million and 2.6 million whole genome Ion PGM sequenced FASTQ reads, respectively, by means of MIRA/MITObim pipeline [46, 47]. MITOS software package [48] was then engaged to annotate the assembled mtDNA sequences, and the coding genes were further validated manually with the aid of an online in silico translation tool [49]. Ambiguous mitogenome sequences were verified by PCR-Sanger sequencing-based approach (BigDye v3.1) with specific primers. Mitochondrial genomes of the twelve sequenced mushroom corals [1, 5] were retrieved from databases and reanalyzed alongside our sequenced species.

### Estimation of mitochondrial transcripts abundance

RNAseq mapping analysis of quality-filtered whole transcriptome Ion PGM sequenced FASTQ reads was applied to estimate mitochondrial gene transcript numbers on CLC genomic workbench v7.5 (CLC-Bio, Aarhus, Denmark). 7,609,490 (*R. yuma*) and 4,717,133 (*A. fenestrafer*) whole transcriptome PGM single reads were independently normalized based on the sizes of individual mtDNA regions [50]. Six replicates were performed for each sample. Splicing efficiencies of ND5 and COI precursor RNA transcripts were simply determined by manually counting the reads that unambiguously flanked splice sites and exon–exon ligation sites of the RNAseq mapped genes and ligated exon sequences of ND5 and COI. The transcript values obtained were compared with respective copy numbers generated from qPCR analysis.

### Validation of transcripts by RT-PCR and RT-qPCR

Reverse transcription PCR (RT-PCR) amplification was implemented using sequence specific primers to investigate: (1) intron RNA circles of ND5-717 and COI-884 with divergent primers, (2) numbers of transcripts flanking splice site and exons ligation junctions of *ND5* and *COI* based on convergent primers, and (3) the back-splicing circular RNA intermediate (MTcircRNA-ND5) using divergent primers (Additional file 7: Table S3). Approximately 300 ng total RNA was subjected to reverse transcription using SuperScript III (ThermoFisher Scientific, Waltham, MA, USA) after treatment with DNase-I enzyme (ArctiZymes, Tromsø, Norway), and then subsequently PCR amplification. Equal amounts of RNA with no reverse transcription were amplified as negative controls alongside the samples. All amplicons were resolved on 1% agarose gel, purified using NucleoSpin Gel, and PCR Clean-up method (MACHEREY–NAGEL, GmbH & Co, Düren, Germany), and Sanger sequenced (BigDye v3.1) on both strands. The splicing efficiencies of ND5-717 and COI-884 intron RNAs were further validated by real time quantitative PCR (RT-qPCR) of the splice sites and exon–exon junctions on LightCycler^®^ 96 Real Time PCR System (Roche Diagnostics GmbH, Mannheim, Germany). The reaction mixtures were prepared using SYBR Green Master FastStart Essential DNA Green Master Kit protocol (Roche Diagnostics GmbH, Mannheim, Germany). A reaction volume of 10 µl comprising 5 µl 2X SYBR Green Master FastStart Essential DNA Green Master, 1 µl specific primers (2.5 µM), 2 µl RNase free water (Roche) and 2 µl cDNA (12.5 ng) was used for each sample. Transcript copy numbers were determined using concentrations generated by mean Cq (quantification cycle) values of the samples obtained by Standard curve analyses based on tenfold dilutions of their respective PCR amplicons of known concentrations.

### Plasmid cloning and direct sequencing of back-splicing circular RNA

The 1.5 kb circRNA amplicons obtained by RT-PCR were cloned into pGEM-T easy vector System I (Promega, Madison, WI, USA). 25 ng of the inserts (amplicons) were ligated with 50 ng pGEM-T overnight at 4 °C, and subsequently transformed into DH5α *E. coli* (ThermoFisher Scientific, Waltham, MA, USA). The cells were harvested, plasmid DNA isolated, and subsequently Sanger sequenced on both strands (BigDye v3.1) using divergent primers (Additional file 7: Table S3). Positive controls with insert DNA (from the pGEM-T easy vector Kit), and background controls without PCR product, were included in the experiment.

## Additional files

**Additional file 1: Table S1.** Annotation of conventional genes and intergenic regions in *Ricordea yuma* and *Amplexidiscus fenestrafer* mtDNAs.

**Additional file 2: Figure S1.** Amino acid sequence alignment of putative antisense open reading frame (aORF) protein encoded within Intergenic Region 11 (IGR-11) of Corallimorpharia mtDNA. (.) and (-) represent identical residue and deleted residue, respectively, compared to that of *Amplexidiscus fenestrafer*. (#) represents stop codon.

**Additional file 3: Figure S2.** RNA mapping and processing of COI-884 and ND5-717 introns in *Ricordea yuma*. (**A**) Ion Torrent PGM read map of unspliced COI precursor RNA. Number of PGM reads that covers the exon–intron junctions are indicated (red numbers 2/24). (**B**) Ion Torrent PGM read map of spliced COI mRNA. Number of PGM reads that covers the ligated exon junction is indicated (red number 86). (**C**) Ion Torrent PGM read map of unspliced ND5 precursor RNA. Number of PGM reads that covers the exon–intron junctions are indicated (red numbers 18/12). (**D**) Ion Torrent PGM read map of spliced ND5 mRNA. Number of PGM reads that covers the ligated exon junction is indicated (red number 6). (**E**) Gel image of PCR amplicons of ligated exon COI mRNA (left) and circle ligation of COI-884 intron RNA (right). (**F**) Gel image of PCR amplicon of ligated exon ND5 mRNA (left). No circle ligation amplicon was detected from ND5-717 intron RNA (right). (**G**) Sequence read (chromatogram) of COI-884 intron circles. The 3′ end of the intron RNA makes circles with intron positions close to the intron 5′ end, which include both full-length intron circles and 5′ truncated circles.

**Additional file 4: Figure S3.** RNA mapping and processing of COI-884 and ND5-717 introns in *Amplexidiscus fenestrafer*. (**A**) Ion Torrent PGM read map of unspliced COI precursor RNA. Number of PGM reads that covers the exon–intron junctions are indicated (red numbers 6/14). (**B**) Ion Torrent PGM read map of spliced COI mRNA. Number of PGM reads that covers the ligated exon junction is indicated (red number 59). (**C**) Ion Torrent PGM read map of unspliced ND5 precursor RNA. Number of PGM reads that covers the exon–intron junctions are indicated (red numbers 9/13). (**D**) Ion Torrent PGM read map of spliced ND5 mRNA. Number of PGM reads that covers the ligated exon junction is indicated (red number 4). (**E**) Gel image of PCR amplicons of ligated exon COI mRNA (left) and circle ligation of COI-884 intron RNA (right). (**F**) Gel image of PCR amplicon of ligated exon ND5 mRNA (left). No circle ligation amplicon was detected from ND5-717 intron RNA (right). (**G**) Sequence read (chromatogram) of COI-884 intron circles. The 3′ end of the intron RNA makes circles with intron positions close to the intron 5′ end, which include both full-length intron circles and 5′ truncated circles.

**Additional file 5: Table S2.** Splicing efficiencies of ND5-717 and COI-884 introns.

**Additional file 6: Figure S4.** *Amplexidiscus fenestrafer* back-splicing ND5 precursor RNA coverage. Ion PGM transcriptome read mapping of predicted precursor RNA.

**Additional file 7: Table S3.** Oligo primer information.

## Data Availability

The mitochondrial genome sequences of *Amplexidiscus fenestrafer* and *Ricordea yuma* have been deposited at GenBank under the accession numbers MH308002 and MH308004, respectively.

## Abbreviations

aORF: antisense open reading frame
cDNA: complementary DNA
COI-884: cytochrome oxidase I gene group I intron
exo-G: noncoded guanosine cofactor
HEG: homing endonuclease gene
IGR: intergenic region
mRNA: messenger RNA
mtDNA: mitochondrial DNA
ND5-717: NADH dehydrogenase 5 gene group I intron
OxPhos: oxidative phosphorylation
P8: paired segment 8
PGM: personal genome sequencing machine
qPCR: quantitative PCR
rRNA: ribosomal RNA
SS: splice site
tRNA: transfer RNA
UiT: Arctic University of Norway

## References

[1] Medina M, Collins AG, Takaoka TL, Kuehl JV, Boore JL (2006). Naked corals: skeleton loss in scleractinia. Proc Natl Acad Sci USA.

[2] Kitahara MV, Lin MF, Foret S, Huttley G, Miller DJ, Chen CA (2014). The ‘naked’ coral hypothesis revisited—evidence for and against scleractinian monophyly. PLoS ONE.

[3] Fautin DG (2016). Catalog to families, genera, and species of orders Actiniaria and Corallimorpharia (Cnidaria: Anthozoa). Zootaxa..

[4] Emblem Å, Karlsen BO, Evertsen J, Johansen SD (2011). Mitogenome rearrangement in the cold-water scleractinian coral *Lophelia pertusa* (Cnidaria, Anthozoa) involves a long-term evolving group I intron. Mol Phylogenet Evol.

[5] Lin MF, Kitahara MV, Luo H, Tracey D, Geller J, Fukami H, Miller DJ, Chen CA (2014). Mitochondrial genome rearrangements in the scleractinia/corallimorpharia complex: implications for coral phylogeny. Genome Biol Evol..

[6] Rodríguez E, Barbeitos M, Brugler MR, Crowley L, Gusmão L, Häussermann V, Grajales A, Daly M (2014). Hidden among sea anemones: the first comprehensive phylogenetic reconstruction of the order Actiniaria (Cnidaria, Anthozoa, Hexacorallia) reveals a novel group of hexacorals. PLoS ONE.

[7] Emblem Å, Okkenhaug S, Weiss ES, Denver DR, Karlsen BO, Moum T, Johansen SD (2014). Sea anemones possess dynamic mitogenome structures. Mol Phylogenet Evol.

[8] Osigus HJ, Eitel M, Bernt M, Donath A, Schierwater B (2013). Mitogenomics at the base of Metazoa. Mol Phylogenet Evol.

[9] Flot JF, Tiller S (2007). The mitochondrial genome of *Pocillopora* (Cnidaria: Scleractinia) contains two variable regions: the putative D-loop and a novel ORF of unknown function. Gene.

[10] Chi SI, Johansen SD (2017). Zoantharian mitochondrial genomes contain unique complex group I introns and highly conserved intergenic regions. Gene.

[11] Chi SI, Urbarova I, Johansen SD (2018). Expression of homing endonuclease gene and insertion-like element in sea anemone mitochondrial genomes: lesson learned from *Anemonia viridis*. Gene.

[12] Beagley CT, Wolstenholme DR (2013). Characterization and localization of mitochondrial DNA-encoded tRNA and nuclear DNA-encoded tRNAs in the sea anemone *Metridium senile*. Curr Genet.

[13] Beagley CT, Okada NA, Wolstenholme DR (1996). Two mitochondrial group I introns in a metazoan, the sea anemone *Metridium senile*: one intron contains genes for subunits 1 and 3 of NADH dehydrogenase. Proc Natl Acad Sci USA.

[14] Cech TR (1990). Self-splicing of group I introns. Ann Rev Biochem..

[15] Hedberg A, Johansen SD (2013). Nuclear group I introns in self-splicing and beyond. Mobile DNA..

[16] Vicens Q, Cech TR (2006). Atomic level architecture of group I introns revealed. Trends Biochem Sci.

[17] Nielsen H, Johansen SD (2009). Group I introns: moving in new directions. RNA Biol.

[18] Goddard MR, Leigh J, Roger AJ, Pemberton AJ (2006). Invasion and persistence of a selfish gene in the Cnidaria. PLoS ONE.

[19] Fukami H, Chen CA, Chiou CY, Knowlton N (2007). Novel group I introns encoding a putative homing endonuclease in the mitochondrial cox1 gene of Scleractinian corals. J Mol Evol.

[20] Celis JS, Edgell DR, Stelbrink B, Wibberg D, Hauffe T, Blom J, Kalinowski J, Wilke T (2017). Evolutionary and biogeographical implications of degraded LAGLIDADG endonuclease functionality and group I intron occurrence in stony corals (Scleractinia) and mushroom corals (Corallimorpharia). PLoS ONE.

[21] Stoddard BL (2005). Homing endonuclease structure and function. Q Rev Biophys.

[22] Lasda E, Parker R (2014). Circular RNAs: diversity of form and function. RNA.

[23] Li M, Ding W, Sun T, Tariq MA, Xu T, Li P, Wang J (2018). Biogenesis of circular RNAs and their roles in cardiovascular development and pathology. FEBS J.

[24] Bach D-H, Lee SK, Sood AK (2019). Circular RNAs in cancer. Mol Ther Nucleic Acids..

[25] Cech TR, Damberger SH, Gutell RR (1994). Representation of the secondary and tertiary structure of group I introns. Nat Struct Biol..

[26] Nielsen H (2012). Group I ribozymes. Methods Mol Biol.

[27] Zhang Y, Zhang XO, Chen T, Xiang JF, Yin QF, Xing YH, Zhu S, Yang L, Chen LL (2013). Circular intronic long noncoding RNAs. Mol Cell.

[28] Li Z, Huang C, Bao C, Chen L, Lin M, Wang X, Zhong G, Yu B, Hu W, Dai L, Zhu P, Chang Z, Wu Q, Zhao Y, Jia Y, Xu P, Liu H, Shan G (2015). Exon-intron circular RNAs regulate transcription in the nucleus. Nat Struct Mol Biol..

[29] Nielsen H, Fiskaa T, Birgisdottir AB, Haugen P, Einvik C, Johansen SD (2003). The ability to form full-length intron RNA circles is a general property of nuclear group I introns. RNA.

[30] Barfod ET, Cech TR (1989). The conserved U.G pair in the 5′ splice-site duplex of a group I intron is required in the 1st but not the 2nd step of self-splicing. Mol Cell Biol..

[31] Burger G, Yan Y, Javadi P, Lang FB (2009). Group I-intron trans-splicing and mRNA editing in the mitochondria of placozoan animals. Trends Genet.

[32] Grewe F, Viehoever P, Weisshaar B, Knoop V (2009). A trans-splicing group I intron and tRNA-hyperediting in the mitochondrial genome of the lycophyte *Isoetes engelmannii*. Nucleic Acids Res.

[33] Dolan GF, Müller UF (2014). Trans-splicing with the group I intron ribozyme from *Azoarcus*. RNA.

[34] Fiskaa T, Birgisdottir AB (2010). RNA reprogramming and repair base don trans-splicing group I ribozymes. N Biotechnol..

[35] Müller UF (2017). Design and experimental evolution of trans-splicing group I intron ribozymes. Molecules.

[36] Dubin A, Chi SI, Emblem Å, Moum T, Johansen SD (2019). Deep-water sea anemone with a two-chromosome mitochondrial genome. Gene.

[37] Puttaraju M, Been MD (1992). Group I permuted intron-exon (PIE) sequences self-splice to produce circular exons. Nucleic Acids Res.

[38] Umekage S, Kikuchi Y (2006). Production of circular form of streptavidin RNA aptamer in vitro. Nucleic Acids Symp Ser.

[39] Umekage S, Uehara T, Fujita Y, Suzuki H, Kikuchi Y, Agbo EC (2012). In vivo circular RNA expression by the permuted intron exon method. Innov Biotechnol.

[40] Kashima T, Rao N, David CJ, Manley JL (2007). hnRNP A1 functions with specificity in repression of SMN2 exon 7 splicing. Hum Mol Genet.

[41] Qu D, May RJ, Sureban SM, Weygant N, Chandrakesan P, Ali N, Li L, Barrett TA, Houchen CW (2014). Inhibition of Notch signaling reduces the number of surviving Dclk1^+^ reserve crypt epithelial stem cells following radiation injury. Am J Physiol Gastrointest Liver Physiol..

[42] Bai Y, Shakeley RM, Attardi G (2000). Tight control of respiration by NADH dehydrogenase ND5 subunit gene expression in mouse mitochondria. Mol Cell Biol.

[43] Chomyn A (2001). Mitochondrial genetic control of assembly and function of complex I in mammalian cells. J Bioenergy Biomembr.

[44] Memczak S, Jens M, Elefsinioti A, Torti F, Krueger J, Rybak A, Maier L, Mackowiak SD, Gregersen LH, Munschauer M, Loewer A, Ziebold U, Landthaler M, Kocks C, Le Noble F, Rajewsky N (2013). Circular RNAs are a large class of animal RNAs with regulatory potency. Nature.

[45] Andreeva K, Cooper NG (2015). Circular RNAs: new players in gene regulation. Adv Biosci Biotechnol.

[46] Chevreux B, Wetter T, Suhai S. Genome sequence assembly using trace signals and additional sequence information. In: Proc German Conf Bioinform, vol. 99. 1999. p. 45–56.

[47] Hahn C, Bachmann L, Chevreux B (2013). Reconstructing mitochondrial genomes directly from genomic next-generation sequencing reads—a baiting and iterative mapping approach. Nucleic Acids Res.

[48] Bernt M, Donath A, Jühling F, Externbrink F, Florentz C, Fritzsch G, Stadler PF (2013). MITOS: improved de novo metazoan mitochondrial genome annotation. Mol Phylogenet Evol.

[49] Bikandi J, San Millán R, Rementeria A, Garaizar J (2004). In silico analysis of complete bacterial genomes: PCR, AFLP-PCR, and endonuclease restriction. Bioinformatics.

[50] Mortazavi A, Williams BA, McCue K, Schaeffer L, Wold B (2008). Mapping and quantifying mammalian transcriptomes by RNA-seq. Nat Methods.

