# Supplementary material for: Giant group I intron in a mitochondrial genome is removed by RNA back-splicing
Source: BMC Mol Biol. 2019 Jun 1;20:16. doi: 10.1186/s12867-019-0134-y (PMC6545197; doi:10.1186/s12867-019-0134-y)
Supplement: Supplementary file 1 — Additional file 1: Table S1. Annotation of conventional genes and intergenic regions in Ricordea yuma and Amplexidiscus fenestrafer mtDNAs. [file 12867_2019_134_MOESM1_ESM.pdf]

Additional file 1: Table S1: Annotation of conventional genes and intergenic regions (IGRs) in *Ricordea yuma* (Ryu) and *Amplexidiscus fenestrafer* (Afe) mtDNAs.

**Genes & IGSs**	**Ryu - Position**	**Size nt**	**Afe - Position**	**Size nt**
ND5-5'	1-720	720 GTG	1-720	720 GTG
I717-5'	721-1157	437	721-1064	344
IGR-1	1158-1318	161	1065-1231	167
M	1319-1389	71	1232-1302	71
IGR-2	1390-1425	36	1303-1328	26
LSU	1426-3820	2395	1329-3657	2329
IGR-3	3821-4033	213	3658-3856	199
ND1	4034-5017	984	3857-4840	984
IGR-4	5018-5237	220	4841-5126	286
CytB	5238-6377	1140	5127-6287	1161
IGR-5	6378-6621	244	6288-6417	130
ND4L	6622-6921	300 GTG	6418-6717	33 GTG
IGR-6	6922-6972	51	6718-6779	62
ND3	6973-7329	357	6780-7136	357 GTG
IGR-7	7330-7670	341	7137-7279	143
A8	7671-7883	213	7280-7489	210
IGR-8	7884-7941	58	7490-7566	77
COI-5'	7942-8837	896	7567-8462	896
I884	8838-10035	1198	8463-9668	1206
HEG	8837-9922	1086	8512-9555	1044
COI-3'	10036-10752	717	9669-10413	745
IGR-9	10753-10844	92	10414-10528	115
COIII	10843-11631	789	10529-11317	789
IGR-10	11632-11682	51	11318-11368	51
COII	11683-12438	756 GTG	11369-12124	756
IGR-11	12439-13366	928	12125-12665	541
ND2	13367-14464	1098	12666-13763	1098
IGR-12	14465-14500	122	13764-13918	155
SSU	14501-15726	1140	13919-15066	1148
IGR-13	15727-16858	1132	15067-15497	431
ND4	16859-18334	1476 GTG	15498-16973	1476 GTG
IGR-14	18335-18374	40	16974-17070	97
ND6	18375-18980	606	17071-17676	606 GTG
IGR-15	18981-19036	56	17677-17744	68
A6	19037-19735	699	17745-18443	699
IGR-16	19736-19944	209	18444-18603	160
I717-3'	19945-20021	77	18604-18680	77
ND5-3'	20022-21140	1119	18681-19799	1119
IGR-17	21141-21221	81	19800-19860	61
W	21222-21291	70	19861-19930	70
IGR-18	21292-21430	139	19931-20054	124
