# Supplementary material for: Giant group I intron in a mitochondrial genome is removed by RNA back-splicing
Source: BMC Mol Biol. 2019 Jun 1;20:16. doi: 10.1186/s12867-019-0134-y (PMC6545197; doi:10.1186/s12867-019-0134-y)
Supplement: Supplementary file 2 — Additional file 2: Figure S1. Amino acid sequence alignment of putative antisense open reading frame (aORF) protein encoded within Intergenic Region 11 (IGR-11) of Corallimorpharia mtDNA. (.) and (-) represent identical residue and deleted residue, respectively, compared to that of Amplexidiscus fenestrafer. (#) represents stop codon. [file 12867_2019_134_MOESM2_ESM.pdf]

Additional file 2: Figure S1. Amino acid sequence alignment of putative antisense open reading frame protein (aORF) encoded within Intergenic Region 11 (IGR-11) of Corallimorpharia mtDNA.

```
Amplexiduscus fenestrafer (Afe)     MVNRAVVVVGAPSYTSGAHIWKGATDNLNRRATTINKIPMGTRAS
Discosoma nummiforme (Dnu)          .T........................S..........T.......
Discosoma sp. (Dsp)                 .......I..................S............I.....
Rhodactis mussoides (Rmu)           .......I..................S............I.....
Rhodactis indosinensis (Rin)        .............................................
Rhodactis sp. (Rsp)                 .............................................
Corynactis californica (Cca)        ...K...A................I....K.......P.I.....
Corallimorphus profundus (Cpr)      IA......................I...KK.......L.I...L.
Pseudocorynactis sp. (Psp)
Ricordea florida (Rfl)
Ricordea yuma (Ryu)

Afe     EREHPELSTKFIYDIWTPPVSPHNKAHPNKNKPEESAPNTKYFKPASALCPDPPPWAANIKRERVDSSM
Dnu     .....................................................................
Dsp     ....................N...R............................................
Rmu     ....................N...R............................................
Rin     .....................................................................
Rsp     .....................................................................
Cca     .KSN.G.......G.......................................................
Cpr     .K#  (47 aa)

Afe     AKKTESQLLEETRQRLPNATIKIKTGKWPFVWKKENFRKTTFPGSTINKTATEPIIIINLAISVQLFII
Dnu     ...........I............................S............................
Dsp     ........................................S............................
Rmu     ........................................S............................
Rin     ........................................S............................
Rsp     ........................................S............................
Cca     ........P......P......S.M.RS............SS..PI.......................
Psp                                                           M..............
Rfl                             M....L..........N............................
Ryu                             M...............N............................

Afe     HNSPHPSTYKKVHQNSNQKERPAPTSPIINSIKAEGLKIKPLMLTNTVPNIKPFKTEKTLIIHSI----
Dnu     ....Y.P...RF.K..KK....T..P.......T..F....F...I.A..V.------AP.M..-----
Dsp     ....Y.P....F.K..KK....T..P.......T..F....F...IAA..V.------A.....----
Rmu     ....Y.P....F.K..KK....T..P.......T..F....F...IAA..V.------A.....RPKFH
Rin     ....Y.P.#  (191 aa)
Rsp     ....Y.P.#  (191 aa)
Cca     ....Y.P.H..F.KS.KK..K.T..P....NT.I..F.P...I..IPA.KV.TP..KNP.....-----
Psp     ....Y.P....F.KS.KK..K.T.A.....N..I..F.T...I..I.A..V.TP..K.S.....-----
Rfl     ....Y.P....F....KK..E.T.......N..I.DF.T...I..S.A..V.TP..K.S.....-----
Ryu     ....Y.P....F....KK..E.T.IT....N..I.DF.T...I..I.A....TP..K.S.....-----

Afe     THKIKLNPGVWAQPLSLFKQIHQETPAPLGRPDIVPNKQLQNRSKPINKGISIFEMSGMN#  (308 aa)
Dnu     .......S.....S......MFH#  (264 aa)
Dsp     ...............P...........S............RS........P....I....#  (302 aa)
Rmu     ...............P...........S............RS........P....I..RT#  (306 aa)
Cca     .R.A.HP#  (254 aa)
Psp     .R....KDLSHK.RSV#  (95 aa)
Rfl     ...S..KTSLINKRMFKFQMTENNPTHQLLHQTQPNINQNKPSTRPQHTPLFKQTHQETPTSLGPANIM
Ryu     ..QT.TKDLSHK.KSV#  (125 aa)

Rfl     PNRPLKH#  (185 aa)
```
