# Supplementary figures and images for: Giant group I intron in a mitochondrial genome is removed by RNA back-splicing
Source: BMC Mol Biol. 2019 Jun 1;20:16. doi: 10.1186/s12867-019-0134-y (PMC6545197; doi:10.1186/s12867-019-0134-y)

Additional file 3: Figure S2

*Ricordea yuma*; COI - 884 (A, B, E, G) and ND5 - 717 (C, D, F)

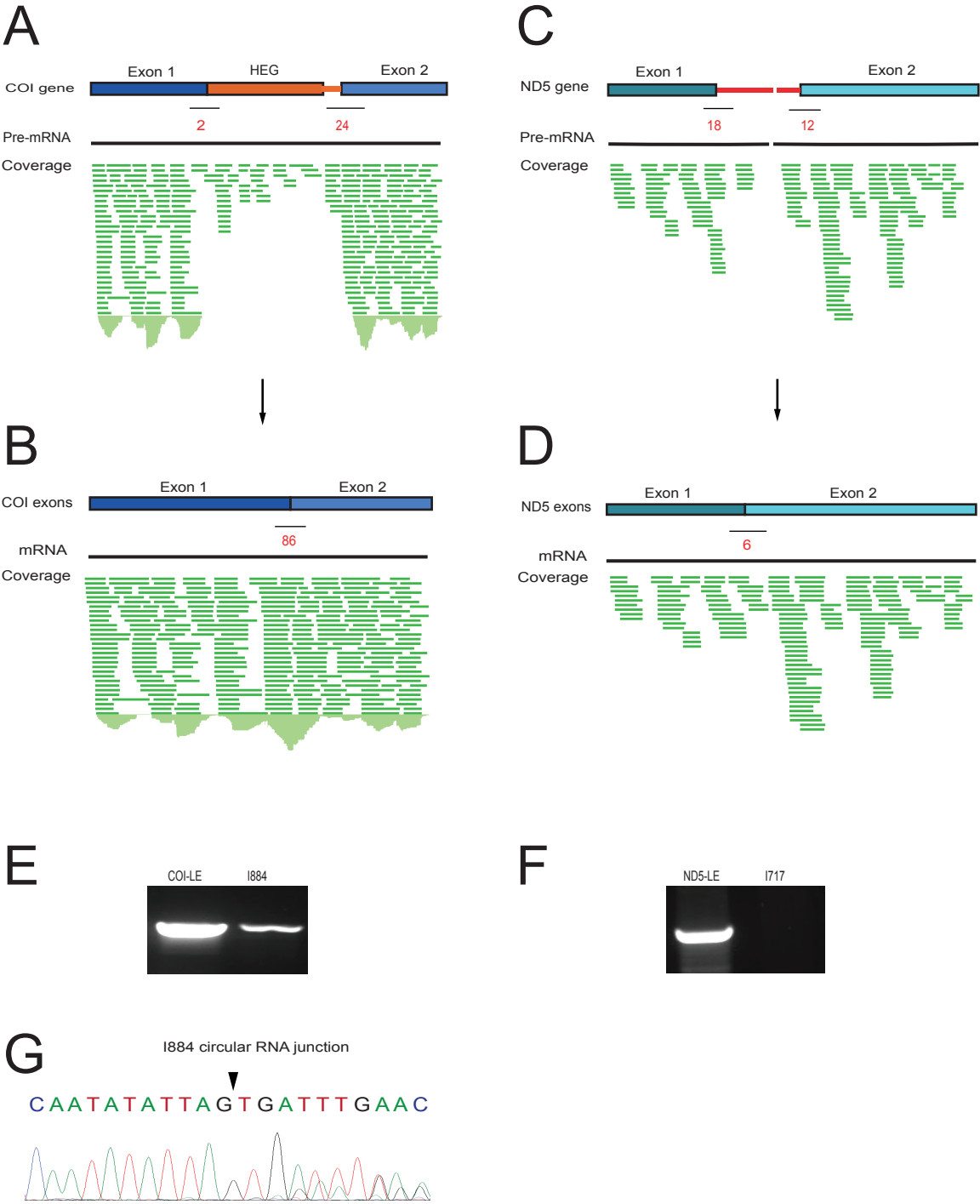

Figure S2

Supplement: Supplementary file 3 — Additional file 3: Figure S2. RNA mapping and processing of COI-884 and ND5-717 introns in Ricordea yuma. (A) Ion Torrent PGM read map of unspliced COI precursor RNA. Number of PGM reads that covers the exon–intron junctions are indicated (red numbers 2/24). (B) Ion Torrent PGM read map of spliced COI mRNA. Number of PGM reads that covers the ligated exon junction is indicated (red number 86). (C) Ion Torrent PGM read map of unspliced ND5 precursor RNA. Number of PGM reads that covers the exon–intron junctions are indicated (red numbers 18/12). (D) Ion Torrent PGM read map of spliced ND5 mRNA. Number of PGM reads that covers the ligated exon junction is indicated (red number 6). (E) Gel image of PCR amplicons of ligated exon COI mRNA (left) and circle ligation of COI-884 intron RNA (right). (F) Gel image of PCR amplicon of ligated exon ND5 mRNA (left). No circle ligation amplicon was detected from ND5-717 intron RNA (right). (G) Sequence read (chromatogram) of COI-884 intron circles. The 3′ end of the intron RNA makes circles with intron positions close to the intron 5′ end, which include both full-length intron circles and 5′ truncated circles. [file 12867_2019_134_MOESM3_ESM.pdf]

Additional file 4: Figure S3

***Amplexodiscus fenestrafer***; COI - 884 (A, B, E, G) and ND5 - 717 (C, D, F)

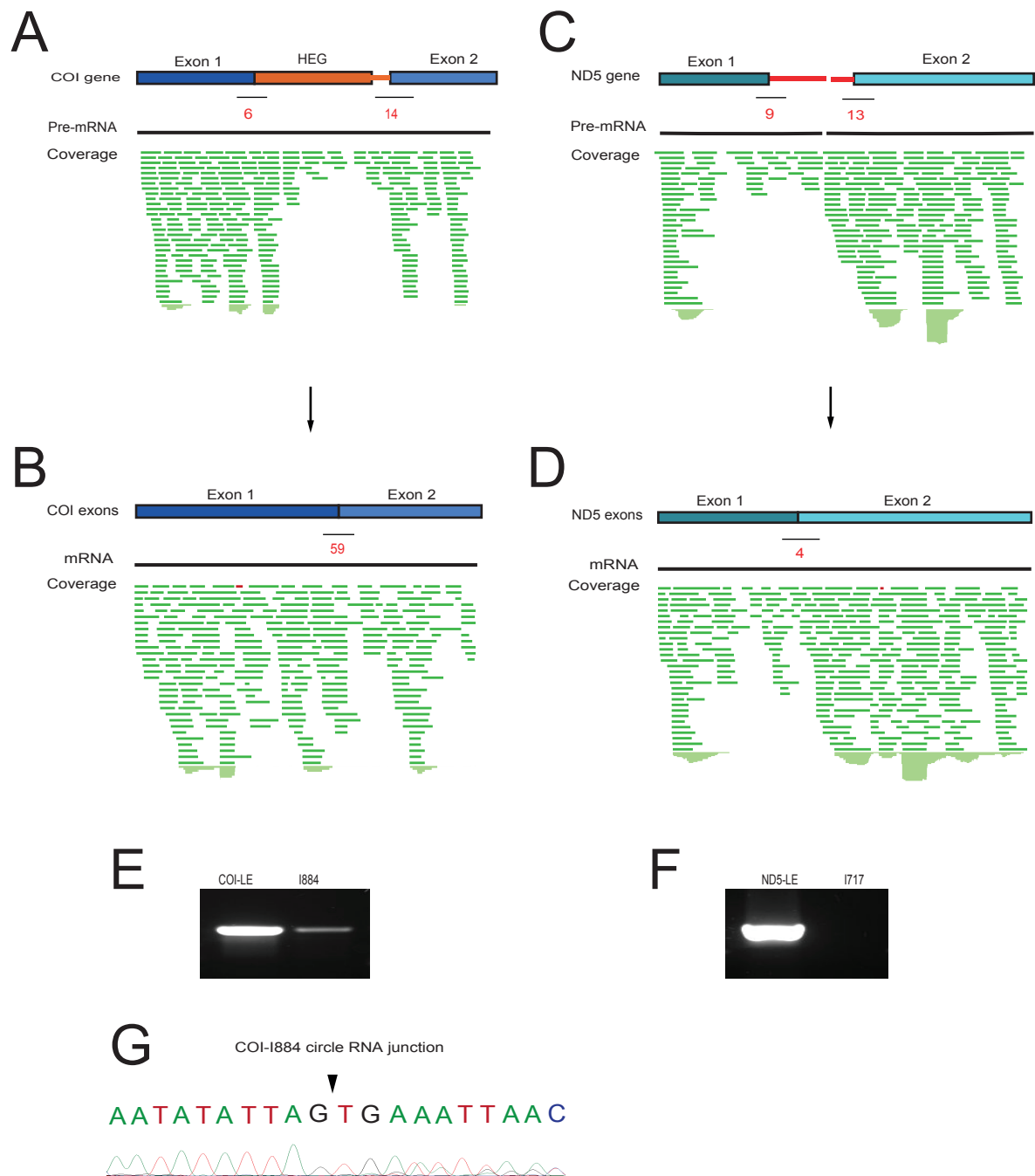

Supplement: Supplementary file 4 — Additional file 4: Figure S3. RNA mapping and processing of COI-884 and ND5-717 introns in Amplexidiscus fenestrafer. (A) Ion Torrent PGM read map of unspliced COI precursor RNA. Number of PGM reads that covers the exon–intron junctions are indicated (red numbers 6/14). (B) Ion Torrent PGM read map of spliced COI mRNA. Number of PGM reads that covers the ligated exon junction is indicated (red number 59). (C) Ion Torrent PGM read map of unspliced ND5 precursor RNA. Number of PGM reads that covers the exon–intron junctions are indicated (red numbers 9/13). (D) Ion Torrent PGM read map of spliced ND5 mRNA. Number of PGM reads that covers the ligated exon junction is indicated (red number 4). (E) Gel image of PCR amplicons of ligated exon COI mRNA (left) and circle ligation of COI-884 intron RNA (right). (F) Gel image of PCR amplicon of ligated exon ND5 mRNA (left). No circle ligation amplicon was detected from ND5-717 intron RNA (right). (G) Sequence read (chromatogram) of COI-884 intron circles. The 3′ end of the intron RNA makes circles with intron positions close to the intron 5′ end, which include both full-length intron circles and 5′ truncated circles. [file 12867_2019_134_MOESM4_ESM.pdf]
