# Supplementary material for: Giant group I intron in a mitochondrial genome is removed by RNA back-splicing
Source: BMC Mol Biol. 2019 Jun 1;20:16. doi: 10.1186/s12867-019-0134-y (PMC6545197; doi:10.1186/s12867-019-0134-y)
Supplement: Supplementary file 5 — Additional file 5: Table S2. Splicing efficiencies of ND5-717 and COI-884 introns. [file 12867_2019_134_MOESM5_ESM.pdf]

Additional file 5: Figure S4

Amplexodiscus fenestrafer back-splicing precursor RNA coverage

ND5 precursor RNA

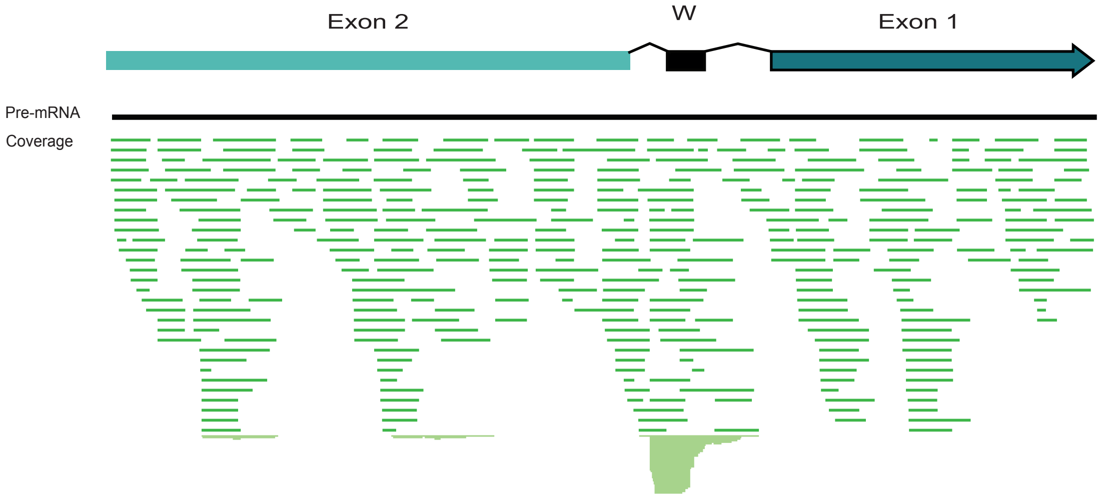

Figure S4
