# Supplementary material for: Giant group I intron in a mitochondrial genome is removed by RNA back-splicing
Source: BMC Mol Biol. 2019 Jun 1;20:16. doi: 10.1186/s12867-019-0134-y (PMC6545197; doi:10.1186/s12867-019-0134-y)
Supplement: Supplementary file 6 — Additional file 6: Figure S4. Amplexidiscus fenestrafer back-splicing ND5 precursor RNA coverage. Ion PGM transcriptome read mapping of predicted precursor RNA. [file 12867_2019_134_MOESM6_ESM.pdf]

Additional file 6: Table S2: Splicing efficiency of ND5-717 and COI-884 introns.

Transcript numbers at the immediate exon-exon junction (mRNA) and 5`splice site (SS) of ND5 and COI genes of *Ricordea yuma* and *Amplexidiscus fenestrafer*, estimated by manual counting of reads and confirmed by qPCR.

	Ricordea yuma			***Amplexidiscus fenestrafer***			
	Manual reads counts	qPCR analysis		Manual reads counts	qPCR analysis		
Gene Region	*Reads # at junction*	*Mean conc. (ng)*	*Transcript copy #*	*Reads # at junction*	*Mean conc. (ng)*	*Transcript copy #*	*Gene Region*
ND5 E1/E2	6	1.78E-02	8.83E+06	4	6.09E-03	3.02E+06	ND5 E1/E2
ND5-717 5`SS	18	1.77E-01	1.39E+08	9	9.31E-03	7.98E+06	ND5 5`SS
**% spliced**	**25**	–	**6**	**31**	–	**27**	**% spliced**
**% unspliced**	**75**	–	**94**	**70**	–	**73**	**% unspliced**
COI E1/E2	86	4.75E-08	2.69E+01	59	8.35E-07	4.64E+02	COI E1/E2
COI-884 5`SS	2	1.63E-16	7.51E-08	6	2.89E-10	1.34E-01	COI 5`SS
**% spliced**	**98**	–	**100**	**91**	–	**100**	**% spliced**
**% unspliced**	**2**	–	**0**	**9**	–	**0**	**% unspliced**
