# Supplementary material for: Giant group I intron in a mitochondrial genome is removed by RNA back-splicing
Source: BMC Mol Biol. 2019 Jun 1;20:16. doi: 10.1186/s12867-019-0134-y (PMC6545197; doi:10.1186/s12867-019-0134-y)
Supplement: Supplementary file 7 — Additional file 7: Table S3. Oligo primer information. [file 12867_2019_134_MOESM7_ESM.pdf]

Additional file 7: Table S3: Oligo primer information.

	Target region	*Primer ID*	*Primer sequence*	*Amplicon size*
R. yuma	MTcircRNA-ND5	OP2040 FP<br>OP2041 RP	5` TTTAGGACACCTCTTTCGTCTATTG 3`<br>5` CCAATTGACTACAAGTCGAATAAGC 3`	1.5 Kb
A. fenestrafer	MTcircRNA-ND5	OP2047 FP<br>OP2048 RP	5` ATGCTCTGCTTGGCAGTTTAACTAT 3`<br>5` ACCATATACCCCAATTGACTACAAG 3`	1.5 Kb
R. yuma/ *A. fenestrafer*	COI exons	OP2257 FP<br>OP2260 RP	5`AGGAATGGTTTATGCAATGCT 3`<br>5`TCCAGTTGGTACAGCAATAATCA 3`	278 bp
R. yuma/ *A. fenestrafer*	COI 5`SS	OP2257 FP<br>OP2258 RP	5`AGGAATGGTTTATGCAATGCT 3`<br>5`CAAGAGGGGAAAAACGAACA 3`	168 bp
R. yuma/ *A. fenestrafer*	COI 3`SS	OP2259 FP<br>OP2260 RP	5`GGTGTTGGGATTAAGGTACAGTC 3`<br>5`TCCAGTTGGTACAGCAATAATCA 3`	151 bp
R. yuma/ *A. fenestrafer*	COI-884 flcDNA	OP2261 FP<br>OP2262 RP	5`TTGGGATTAAGGTACAGTCCAG 3`<br>5`TTCCCAAAAGATTGGCTCAG 3`	261 bp
R. yuma/ *A. fenestrafer*	ND5 exons	OP1970 FP<br>OP1971 RP	5`TGGAAAATCTGCACAGTTGG 3`<br>5`GGCTTGCTCTAAAAAGGGAGA 3`	995 bp
R. yuma/ *A. fenestrafer*	ND5 5`SS	OP2249 FP<br>OP2250 RP	5`TGGAAAATCTGCACAGTTGG 3`<br>5`CAAGCCTTCCCAGCGTATAG 3`	123 bp
R. yuma/ *A. fenestrafer*	ND5 3`SS	OP2251 FP<br>OP2252 RP	5` TCTATTATGAGAGTAGTAGTGGGCTGA 3`<br>5`GGCTTGCTCTAAAAAGGGAGA 3`	145 bp
R. yuma/ *A. fenestrafer*	ND5-717 flcDNA	OP1918 FP<br>OP1919 RP	5`TGTATTGGGGGCTGATTTTT 3`<br>5`CCAGTTTGTTACCCGCTGAT 3`	197 bp
